# Reciprocal Relationship between Internet Addiction and Network-Related Maladaptive Cognition among Chinese College Freshmen: A Longitudinal Cross-Lagged Analysis

**DOI:** 10.3389/fpsyg.2017.01047

**Published:** 2017-06-22

**Authors:** Piguo Han, Peng Wang, Qingnan Lin, Yu Tian, Fengqiang Gao, Yingmin Chen

**Affiliations:** ^1^School of Psychology, Shandong Normal UniversityJinan, China; ^2^Department of Preschool Education, Heze UniversityHeze, China

**Keywords:** Internet addiction, network-related maladaptive cognition, college freshmen, cross-lagged panel survey, Chinese

## Abstract

This study explored the reciprocal relationship between Internet addiction (IA) and network-related maladaptive cognition (NMC) in Chinese college freshmen. A short-term longitudinal survey with a sample of 213 college freshmen was conducted in Shandong province, China. The results revealed that IA can significantly predict the generation and development of NMCs, and that when such maladaptive cognitions have been established, they can further adversely affect the extent of the students’ IA. A vicious cycle was observed between these two variables, with IA having predictive priority in its relationship with NMC. This study also determined that the relationship between these two variables was the same for both males and females; therefore, the final model we established can be extensively applied to Chinese college freshmen, regardless of gender. Understanding the reciprocal relationship between these two variables can assist in interventions in IA at the outset of students’ college life.

## Introduction

Since its inception in the 1990s, the Internet has gradually become an integral part of daily life in China, particularly among adolescents aged 10–21 years old (Daniel et al., 2012; Liu et al., 2012). According to the *36th Statistical Report on Internet Development in China*, which was published by the China Internet Network Information Center (CNNIC), the number of adolescent Internet users in China has increased rapidly from 120 million in 2002 to 287 million in 2016 (Tian et al., 2017).

The Internet has produced numerous benefits such as enhanced social connection and well-being (Bessière et al., 2008; Young and de Abreu, 2011). However, Internet addiction (IA), which is characterized by excessive or compulsive Internet use (Young et al., 1999; Shek et al., 2013; Yang et al., 2014) has had numerous negative effects (Joseph et al., 2016). A number of studies have demonstrated that IA can adversely affect physical and mental health (Ayas and Horzum, 2013; Georgios et al., 2014; Mike and Zhong, 2014). For example, adolescents with IA usually experience anxiety, depression, loneliness, low self-esteem, and poor interpersonal relationships (Tokunaga and Rains, 2010; Georgios et al., 2014; Mike and Zhong, 2014), which can further negatively affect their well-being (Tokunaga and Rains, 2010; Georgios et al., 2014; Mike and Zhong, 2014) and academic development (Chuang, 2006; Kim et al., 2008; Tsai et al., 2009; Ahmadi and Saghafi, 2013). Therefore, studying IA in adolescents has critical educational and social implications.

### Relationship between IA and NMC

**FIGURE 1:**
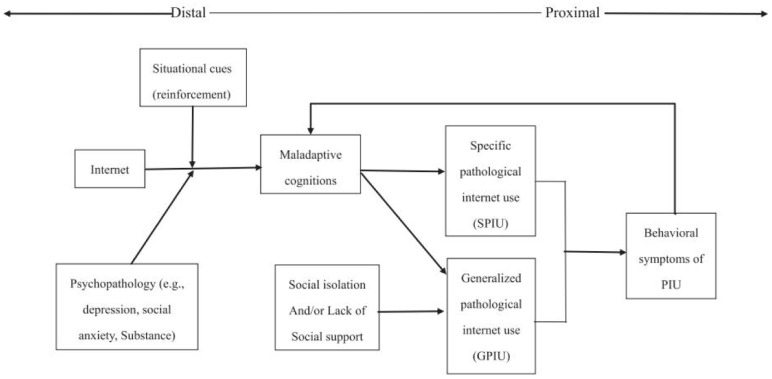
Cognitive-behavioral model of pathological Internet use (Davis, 2001).

Network-related maladaptive cognition (NMC) has long been thought to play a central role in IA (Li et al., 2013). According to the cognitive–behavioral model (Davis, 2001), psychopathology (e.g., depression and social anxiety) is a distal necessary cause of symptoms of IA that does not in itself result in symptoms of IA. The key factors in IA are NMCs, which are proximal sufficient causes (Daniel et al., 2012; **Figure 1**). Numerous studies have reported that distal psychopathology renders an individual vulnerable to IA through NMC (Kalkan, 2012; Mai et al., 2012; Li and Wang, 2013; Lu and Yeo, 2015). For example, researchers investigated the underlying relationship between temperament (i.e., effort control, high sensation seeking, and high dispositional anger or frustration) and the development of IA; the results indicated that certain temperaments influence the level of IA through the effect temperament has on their cognition of online behaviors (Zhang et al., 2015). Tian et al. (2017) examined the reciprocal associations among shyness, maladaptive cognitions, and generalized pathological Internet use (GPIU) in a Chinese sample. The results indicated that the associations among these variables are dynamic and bidirectional, and that the increased maladaptive cognitions bidirectionally mediated the relation between shyness and GPIU across time. Moreover, other studies have determined that parenting style and peer relationship might predispose people to NMC, which would further affect the level of IA (Li et al., 2013; Wang et al., 2015).

In addition, numerous other studies have focused on Internet game addiction and investigated its relationship with maladaptive cognitions. King and Delfabbro (2014) proposed a new model that offers theoretical explanations of the origins and pathogenesis of addiction to Internet games. The authors identified four maladaptive cognitions underlying Internet game addiction, namely overvaluing, maladaptive rules, gaming self-esteem, and gaming acceptance. Some empirical studies also have found that adolescents with Internet game addiction symptoms reported significantly more maladaptive cognitions than adolescents without these symptoms (Zhou et al., 2012; Liu et al., 2014; King and Delfabbro, 2016). Peng and Liu (2010) reported that a five-item scale measuring cognitions significantly predicted Internet gaming addiction in Chinese adults. Forrest et al. (2016) investigated maladaptive cognitions associated with problematic video-game playing in a sample of 465 Australian adults. The results revealed that these problematic cognitions correlated moderately to highly with Internet game addiction. Forrest et al. (2017) explored whether maladaptive cognition could predict future changes in problematic video-gaming using a 12-month longitudinal study. The results showed that cognitive change accounted for 28% of variance in problematic gaming scores beyond gender, age, and frequency of gaming.

Although a number of studies have identified the influence of NMC on IA, few studies have explored the possible influence of IA on NMC. Cognitive dissonance theory (Festinger, 1957), which mainly concerns how people experience and respond to inconsistencies within thinking and between behavior and thinking, provides an alternative explanation of how NMC relates to IA. When people become aware of inconsistencies, they experience discomfort or dissonance, which prompts efforts to reduce these experiences and regain consistency through adapting their attitudes, perceptions, or behaviors until such inconsistencies are resolved (de Vries and Timmins, 2016). According to this theory, when people behave inconsistently with their values, such as by indulging in the Internet when it has already negatively affected their life, they experience dissonance in the form of regret; this occurs with feelings of personal responsibility for the negative consequences of their behaviors. Most people are able to successfully adjust their behavior to reduce this dissonance. However, some people may reduce dissonance by changing their attitude toward the Internet, thereby reducing their dissonance while maintaining problematic behaviors. Chiou and Wan (2007) investigated this process with a sample of video-game players. The results revealed that players who feel responsible for their behavior are more likely to shift their attitudes toward video-games from positive to negative, whereas players with a higher investment in video-game playing are less likely to engage in attitude-discrepant behavior.

### Research on IA among College Students

A variety of studies have suggested that adolescents constitute the majority of Internet users, and that college students are particularly vulnerable to IA because of easy access to the Internet, flexible schedules, and their lower ability to control their behavior (Shaw and Black, 2008; Fu et al., 2010; Georgios et al., 2014; Yang et al., 2014). Moreover, the beginning of college life is a developmental period for students during the transition away from a relatively high reliance on interpersonal relationships (not only family relationships but also peer and other social relationships; Woodhouse et al., 2012). Previous studies have reported that uncontrolled Internet use is closely related to a decline in family communication and supervision (Van den Eijnden et al., 2010; Liu et al., 2012). Thus, the shift in interpersonal relationships tends to place college freshmen at risk of developing IA (Zhang et al., 2014).

In addition, freshmen must pass a series of rigorous exams to gain admission to college in China, and typically have insufficient time for self-reflection during senior high school. Thus, when confronted with college life, their lack of study and interpersonal skills may cause them to feel confused (Ni et al., 2009). Furthermore, owing to abundant leisure time and unlimited Internet access through a range of wireless tools, college freshmen tend to spend large amounts of time online, and are therefore very likely to experience symptoms of IA during this unique period (Chen, 2012). To provide preventive and interventional strategies for IA, a short-term longitudinal study was conducted during a first semester at college.

## The Present Study

**FIGURE 2:**
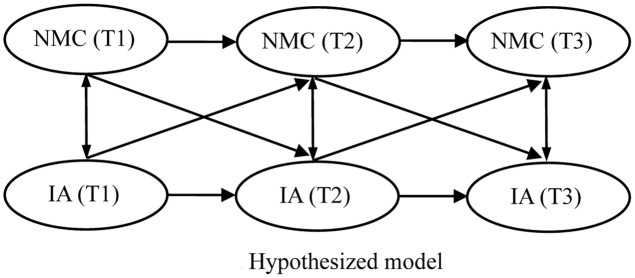
Hypothesized model.

Although many researchers have investigated the relationship between IA and NMC, most have adopted a cross-sectional approach; thus, identifying a reciprocal relationship between these two variables is difficult (Joseph et al., 2016). In addition, although the probability of students developing IA is substantially higher at the beginning of college (Li and Liang, 2007; Ni et al., 2009), the participants of previous studies have typically been college students of all ages, with students in their first semester being involved less often. Therefore, the present study adopted a cross-lagged analysis to explore the relationship between IA and NMC among college students during the first semester of their college life. According to previous theoretical and empirical studies, a reciprocal relationship may exist between these two variables. Therefore, we proposed an interaction model (**Figure 2**) and tested three hypotheses concerning the relationship between these two variables.

H1. Positive and significant cross-lagged paths exist from IA to NMC, and NMC is an effective predictor of IA among Chinese college freshmen.

H2. The cross-lagged paths from IA to NMC reach a significant level, and the level of IA consequently adversely affects NMC.

H3. The relationship between IA and NMC can be generally applied across males and females.

## Materials and Methods

### Participants

The initial number of college freshmen available for participation was 300 participants, all of whom were enrolled in two colleges located in two cities (namely Jinan and Heze) in Shandong province, China. Data collections were conducted in early September of 2015 (T1), 2 months later (T2), and then 4 months later (T3). At the first wave of data collection, all of these 300 students completed the measurement. However, at the subsequent waves, 87 of these 300 students withdrew midway. Lack of participation was due to absence or sickness (participation rate: 71.00%). Therefore, 213 students remained for the final data analysis (104 males and 109 females), with ages ranging from 17 to 21 years (*M* = 18.87 years, *SD* = 0.76 years). In order to determine whether the data of students who withdrew midway (87 students) differed from those who did not withdrew (213 students) with respect to any of the variables included in this study, a series of *t*-tests were conducted using the data collected at the first wave of data collection; none of these analyses were significant. All of these participants had Internet experience and were included in this study. Participants had used the Internet for an average of 5.59 years (*SD* = 2.06) at the beginning of their college life. Information was collected on students’ registered residence: 43.19% lived in large cities, 35.68% lived in towns, and 21.13% lived in villages. In addition, a file was established for each student (these files included their basic information, as well as physical and mental health status) when they entered the college. According to the files, none of the participants have any psychiatric or neurological disorders. This study was carried out in accordance with the recommendations of Shandong Normal University ethical guidelines and the Declaration of Helsinki, with written informed consent from all participants. The protocol was approved by the Human Research Ethics Committee of Shandong Normal University.

### Instruments

#### Internet Addiction

The current study adopted the revised Chinese Internet Addiction Scale (CIAS-R; Bai and Fan, 2005). The CIAS-R contains 19 items that can be divided into four factors: compulsive use and withdrawal (e.g., “I feel depressed during a period of time without Internet access”), tolerance (e.g., “I find myself having to spend increasing amounts of time online to feel satisfied”), time management problems (e.g., “My academic or job performance suffers adverse effects because of my Internet use”), and interpersonal and health problems (e.g., “I reduce my sleeping time to have more time online”). Each response was measured on a 4-point Likert-type scale with scores ranging from 1 (*not at all true*) to 4 (*always true*). Therefore, higher mean scores represent higher levels of IA. The scale has been applied in recent studies on Chinese college students and demonstrated high reliability and validity (Tian et al., 2015). In the present study, the alpha coefficients for the scale were 0.92 at T1, 0.95 at T2, and 0.91 at T3.

#### Network-Related Maladaptive Cognition

This study adopted the Network-related Maladaptive Cognition Scale revised by Liang; the original scale was the Online Cognition Scale, which was developed on the basis of the cognitive–behavioral model proposed by Davis (Tian et al., 2015). The revised scale contains 14 items that can be divided into three factors: Internet comfort (e.g., “I receive more respect online than in ‘real life”’), diminished impulse control (e.g., “When I am on the Internet, I often feel a kind of ‘rush’ or emotional high”), and distraction (e.g., “Using the Internet is a way to forget about the things I must do but really don’t want to do”). Participants rated how true each statement was on a 5-point Likert-type scale, with scores ranging from 1 (*not at all true*) to 5 (*always true*). Hence, higher mean scores represent higher levels of NMC regarding Internet use. The scale has been applied in previous studies on Chinese college students (Tian et al., 2015, 2017). In the current study, the alpha coefficients for the scale were 0.87 at T1, 0.90 at T2, and 0.90 at T3.

### Statistical Analysis

**FIGURE 3:**
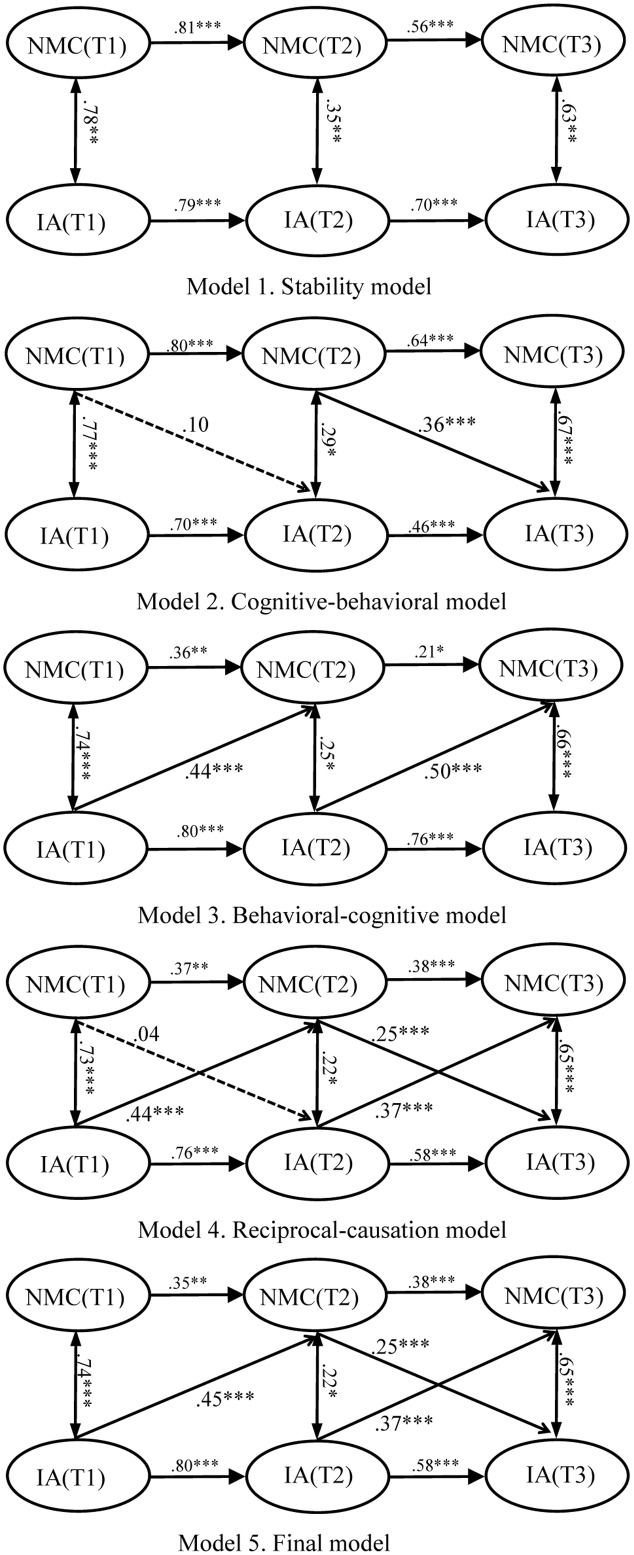
Results from cross-lagged analysis. Single-arrowed lines represent path coefficients and double-arrowed lines represent covariances. Dashed lines indicate non-significant coefficients, and solid lines indicate significant coefficients. ^∗∗∗^indicate coefficient is significant at 0.001 level, ^∗∗^indicate coefficient is significant at 0.01 level, and ^∗^indicate coefficient is significant at 0.05 level.

In this study, we employed a fully cross-lagged panel design to examine the unidirectional and bidirectional relationships between IA and NMC in Chinese college freshmen (Van Lier et al., 2012). The general model consisted of measures of IA and NMC at T1, T2, and T3. We proposed and tested four models representing the possible mechanisms between the two variables. First, we proposed a “stability model” (Model 1, **Figure 3**) that included only cross-time stability effects. Second, a cognitive–behavioral model (Model 2, **Figure 3**) was proposed to examine whether NMC at one time point could predict IA at the following time point. Third, we proposed a “behavioral–cognitive model” (Model 3, **Figure 3**) to examine whether IA at one time point could predict NMC at the following time point. Finally, we proposed a “reciprocal-causation model” (Model 4, **Figure 3**) that explored the reciprocal influence between IA and NMC. In addition, a multigroup cross-lagged analysis based on gender was conducted to examine whether the relationship between the two key variables differed between males and females.

Structural equation modeling with latent variables was employed to test the hypothesized model in this study. In accordance with the recommendation of Holbert and Stephenson (2002), the goodness of model fit was evaluated using a variety of indices. The χ^2^ estimate with degrees of freedom is still the most commonly used means of performing comparisons across different models. The ratio between χ^2^ and degrees of freedom should not exceed 5 for models with a good fit. Additionally, we reported the comparative fit index (CFI) along with the Tucker–Lewis index (TLI) and the root mean square error of approximation (RMSEA). In general, CFI and TLI values of 0.95 or higher reflect good fit and RMSEA values lower than 0.06 indicate excellent fit, whereas values between 0.06 and 0.08 indicate good fit (Yuan et al., 2014). Moreover, the χ^2^ test of difference (Δχ^2^) was used to compare the fit of the nested models. A non-significant Δχ^2^ test indicates that the two models provide an equal fit to the data, whereas a significant Δχ^2^ suggests that the less constrained model should be retained (Tian et al., 2017).

## Results

### Descriptive Analysis

The means and standard deviations of the key variables in this study are presented in **Table 1**. Repeated measures ANOVA was conducted to explore the influences of gender and measurement time on the participants’ IA and NMC (“gender” is a between-subjects variable, and “measurement time” is a within-subjects variable). The results revealed no significant difference between genders in terms of the two dependent variables (*F* = 0.10, *p* = 0.749; *F* = 0.02, *p* = 0.822). Over time, the level of IA significantly increased from T1 to T3 among the college freshmen (*F* = 28.71, *p* < 0.001). The results of a *post hoc* test indicated that the level of IA measured at T3 was significantly higher than those at T2 (*p* < 0.01) and T1 (*p* < 0.001), and that the level of IA measured at T2 was significantly higher than that at T1 (*p* < 0.001). In addition, marginal significant differences existed in NMC when measured at different times (*F* = 2.93, *p* = 0.055). The results of the *post hoc* test revealed that the level of IA measured at T3 was significantly higher than that at T1 (*p* < 0.05), and that marginal significant differences existed in NMC when measured at T1 and T2 (*p* = 0.065). However, no significant difference existed in NMC when measured at T2 and T3 (*p* = 0.846). The interaction between gender and measurement time in the two variables did not reach significant levels (*F* = 0.38, *p* = 0.682; *F* = 0.24, *p* = 0.791).

**Table 1:** Descriptive statistics of IA and NMC (*n* = 213).

	Tl		T2		T3	
	Males	Females	Males	Females	Males	Females
IA	1.90 (0.53)	1.89 (0.58)	2.02 (0.60)	2.07 (0.64)	2.13 (0.60)	2.16 (0.68)
NMC	2.42 (0.66)	2.40 (0.68)	2.49 (0.64)	2.52 (0.71)	2.48 (0.69)	2.51 (0.71)

As shown in **Table 2**, the bivariate correlations between IA and NMC at T1, T2, and T3, as well as all cross-lagged correlations between the two variables, were significant and positive, suggesting a positive relationship between IA and NMC.

**Table 2:** Correlations between IA and NMC (*n* = 213).

	1	2	3	4	5	6
1 I A (T1)	1					
2 I A (T2)	0.71^∗∗^	1				
3 I A (T3)	0.64^∗∗^	0.71^∗∗^	1			
4 NMC (T1)	0.60^∗∗^	0.49^∗∗^	0.42^∗∗^	1		
5 NMC (T2)	0.62^∗∗^	0.59^∗∗^	0.53^∗∗^	0.60^∗∗^	1	
6 NMC (T3)	0.54^∗∗^	0.48^∗∗^	0.67^∗∗^	0.47^∗∗^	0.54^∗∗^	1

*^∗∗^Correlation is significant at the 0.01 level (2-tailed).*

### Cross-Lagged Relationships between IA and NMC

A series of cross-lagged models was specified to examine the reciprocal relationships between IA and NMC. First, a baseline model (Model 1, **Figure 3**) was specified; in this model, the stability coefficients for IA and NMC were estimated, but the cross-lagged effects between the two variables were not estimated. The model fit was acceptable (**Table 3**). Second, to test the cognitive–behavioral model presented previously, the cross-lagged paths from NMC to IA were added to the baseline model (Model 2, **Figure 3**), which improved the model fit significantly (**Table 3**). The χ^2^ test of difference revealed that Model 2 demonstrated a better fit to the data than Model 1 (Δχ^2^ = 27.05, Δ*df* = 2, Δχ^2^/Δ*df* = 13.53 > 6.63) (Wen et al., 2006). According to Model 2, the standardized path coefficients were 0.10 (*p* = 0.309) for NMC measured at T1 to IA measured at T2, and 0.36 (*p* < 0.001) for NMC measured at T2 to IA measured at T3. Third, to examine whether IA at one time point could predict NMC at the following time point, the cross-lagged paths from IA to NMC were added to the baseline model (Model 3, **Figure 3**). The result showed that a good model fit was achieved (**Table 3**). The χ^2^ test of difference demonstrated that Model 3 demonstrated a superior fit to the data than Model 1 did (Δχ^2^ = 47.20, Δ*df* = 2, Δχ^2^/Δ*df* = 23.60 > 6.63). According to Model 3, the standardized path coefficients were 0.44 (*p* < 0.001) for IA measured at T1 to NMC measured at T2, and 0.50 (*p* < 0.001) for IA measured at T2 to NMC measured at T3. This indicates that IA at one time point was an effective predictor of NMC at the following time point, and that the addition of the two paths to the model could improve the model fit significantly. Fourth, Model 4 was specified with both stability coefficients and the cross-lagged effect between IA and NMC (Model 4, **Figure 3**). The model fit the data sufficiently (**Table 3**). However, Models 3 and 4 are nested, and the χ^2^ test of difference indicated that the two models performed equally well (Δχ^2^ = 11.69, Δ*df* = 2, Δχ^2^/Δ*df* = 5.85 < 6.63). Finally, as shown in Model 4, except for the standardized path coefficients for NMC measured at T1 to IA measured at T2, the rest of the cross-lagged paths between IA and NMC reached a significant level. Therefore, we deleted this path and developed Model 5. The model fit the data sufficiently (**Table 3**). Consequently, Model 5 was retained as the final model for analysis because of the following reasons: (1) Although the two models performed equally well (Δχ^2^ = 0.21, Δ*df* = 1, Δχ^2^/Δ*df* = 0.21 < 6.63), Model 5 is simpler and more parsimonious than Model 4 is, and fewer parameters should be selected for analysis. (2) The χ^2^ test of difference showed that Model 5 demonstrated a better fit to the data than Model 3 did (Δχ^2^ = 11.48, Δ*df* = 1, Δχ^2^/Δ*df* = 11.48 > 6.63), and the standardized path coefficient was 0.25 (*p* < 0.001) for NMC measured at T2 to IA measured at T3. That is, a strong possibility exists that NMC measured at T2 can predict IA measured at T3.

**Table 3:** Comparisons between different models.

	χ^2^	*df*	χ^2^/*df*	*CFI*	*TLI*	*RMSEA*
Model 1	382.03	162	2.36	0.94	0.92	0.080
Model 2	354.98	160	2.22	0.95	0.93	0.076
Model 3	334.83	160	2.09	0.95	0.94	0.072
Model 4	323.14	158	2.05	0.96	0.94	0.070
Model 5	323.35	159	2.03	0.96	0.95	0.070

### Gender Differences

To investigate whether the cross-lagged relationships between IA and NMC differ across males and females, we conducted a multigroup analysis. We first estimated the model fit for males (M_male_) and females (M_female_) separately, and the fit indices were adequate for both subsamples (**Table 4**). Measurement invariance was then tested to determine whether both variables were measured identically for males and females. In the fully unconstrained measurement model (M_1_), all parameters were allowed to vary across the two groups. An acceptable model fit was achieved, and a fully constrained measurement model (M_2_) was then analyzed in which all parameters were fixed identically for the two groups; the model fit the data sufficiently (**Table 4**). The χ^2^ test of difference indicated that the two models performed equally well (Δχ^2^ = 6.50, Δ*df* = 15, *p* = 0.970).

**Table 4:** Multigroup analysis across males and females.

	χ^2^	*df*	χ^2^/*df*	*CFI*	*TLI*	*RMSEA*	Δχ^2^(Δ*df*)
M_male_	218.00^∗∗^	159	1.37	0.97	0.95	0.060	—
F_female_	187.56^+^	159	1.18	0.99	0.98	0.041	—
M**l**	405.57^∗∗^	318	1.28	0.98	0.97	0.036	—
M2	412.07^∗∗^	333	1.24	0.98	0.97	0.034	6.50 (15)
M3	413.85^∗∗^	340	1.22	0.98	0.97	0.032	1.78 (7)

*^+^Significant at the 0.1 level (2-tailed); ^∗∗^significant at the 0.01 level (2-tailed).*

To address the research aim of this part of the study, we conducted a multigroup cross-lagged analysis according to the gender of the college students. Three parameters were tested to examine whether they differed between genders: stability coefficients, cross-lagged path coefficients, and covariances between IA and NMC. A constrained model (M_3_) was specified in which all three parameters were identical across the two groups; this model achieved a satisfactory model fit (**Table 4**). The χ^2^ test of difference indicated that this model fit the data as adequately as the fully unconstrained model (Δχ^2^ = 1.78, Δ*df* = 7, *p* = 0.996), indicating that the overall pattern of paths was invariant between males and females.

## Discussion

This study entailed conducting a cross-lagged panel survey to explore the reciprocal relationship between IA and NMC among Chinese college freshmen. According to the cognitive–behavioral model (Davis, 2001), a reciprocal relationship may exist between the two variables, and NMC is a likely predictor of IA. However, this hypothesis was not fully supported. During the first 2 months of the study, we observed no predictive effect of NMC on IA; this is not consistent with the results of a previous study (Tian et al., 2015). Specifically, NMC appears not to be the essential condition for the generation of IA. This result is likely related to the participants in the current study. In this study, the participants of the survey were Chinese college freshmen who had recently completed a strict entrance examination, namely “Gaokao.” To gain admission to college, students must exert great effort throughout their elementary and secondary education; consequently, few of them have ample time to use the Internet (Li and Liang, 2007). Therefore, the level of NMC was lowest when participants enrolled in college, which might have prevented a significant influence on the generation of IA. During the college period, numerous other factors can cause people to become addicted to the Internet. For example, the anonymity and absence of non-verbal and demographic cues provided by the Internet can be beneficial to students’ well-being through its offering relief from emotional distress (Caplan and Turner, 2007) and enhancing perceptions of social support and self-esteem (Kraut et al., 2002), as well as expanding the range of interpersonal relationships (Cotten, 2008). In addition, personality traits can play a critical role in the generation of IA during this period (Mike et al., 2014). For instance, people with high effort control ability were more effective at suppressing impulsive acts when tempted with the Internet, as mentioned previously. By contrast, people with high sensation-seeking levels were more prone to develop addictive behaviors (Zhang et al., 2015). However, the academic stress of Chinese freshmen decreased significantly after entering college, and they had more time to use the Internet (Li and Liang, 2007). Thus, they may have gradually developed various cognitive responses toward the Internet through their own experiences or experiences involving peer exchange, which consequently affected their IA level (Wang et al., 2015).

According to the cognitive–behavioral model (Davis, 2001), IA could have a negative impact on NMC (Caplan, 2010). However, few empirical studies have been conducted to test this hypothesis, and few researchers have proposed theories to explain this phenomenon. Nevertheless, the empirical evidence of the current study suggests that IA had predictive priority in its relationship with NMC, which could affect both the generation and development of NMC. Some researchers have explored online gaming behaviors in Chinese adolescents. They have reported that online gaming behaviors could significantly affect cognition regarding online games because the changing of relative cognitions regarding online games was an efficient method for relieving the discomfort caused by the inconsistencies between behavior and thinking (Wang et al., 2015). Therefore, we applied cognitive dissonance theory as an explanatory model to discuss the predictive-effect mechanism of IA on NMC (Cooper, 2007). According to this theory, when people become aware of inconsistencies, they experience discomfort or dissonance, which prompts efforts to reduce this experience and regain consistency by adapting their attitudes, perceptions, or behaviors until such inconsistencies are resolved (de Vries and Timmins, 2016). Most people may be able to successfully adjust their behavior to reduce this dissonance. However, some people tend to justify behaviors that appear to reduce dissonance, using reasons such as “I can receive more respect online than ‘in real life’,” “I feel safest when on the Internet,” or “Using the Internet is a way to forget about the things I must do but do not wish to do.” When people have convinced themselves that being addicted to the Internet is reasonable, dissonance is reduced and they feel better. However, the effective method for reducing dissonance discomfort tends to be repeated when another identical violation occurs, which is problematic (de Vries and Timmins, 2016). That is, when such NMCs have been established by the students, the subsequent excessive use of the Internet does not produce the same level of discomfort, which further increases excessive Internet use. In summary, the generation and development of IA is caused by a vicious cycle involving NMC and excessive use of the Internet, and IA has the predictive priority in its relationship with NMC.

The results indicate no significant differences in the key variables between males and females. This is inconsistent with the findings of many other studies **(**Müller et al., 2014). This is possibly because of the rapid development of computing and networking technologies, which have considerably changed network terminal equipment as well as their applications (Daniel et al., 2012; Mike and Zhong, 2014). For example, in contemporary society, mobile phones have gradually become the primary means of accessing the Internet, and a variety of activities, such as shopping and browsing, can be performed with them. Both males and females enjoy the majority of such activities (San, 2015). In addition, the results of the multigroup cross-lagged analysis reveal that the paths found among IA and NMC were the same for males and females. That is, the processes leading to IA can be identical for both genders, and the final model established in the present study thus possesses extensive applicability and practical significance for Chinese college freshmen.

**FIGURE 4:**
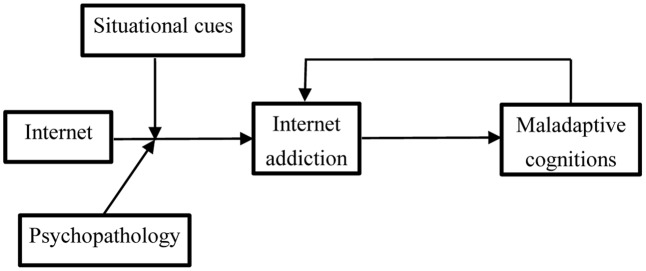
Theoretical model of the present study.

To further identify the generative and developmental mechanism of IA, we proposed a theoretical model based on the results found in this study as well as the cognitive–behavioral model proposed by Davis (**Figure 4**). According to this model, the generation and development of IA is a result of a vicious cycle involving IA and NMC, and this cycle is mainly induced by the discomfort caused by the inconsistencies between behavior and thinking (de Vries and Timmins, 2016). Because this cycle mainly begins with excessive use of the Internet, mitigating this vicious cycle at the beginning of the semester through various methods is necessary. However, because of the powerful appeal of the Internet to young people, completely avoiding IA among college freshmen is difficult. According to cognitive dissonance theory, when some of them become addicted to the Internet, two methods exist for reducing the discomfort caused by the inconsistencies between behavior and thinking. The first method involves changing online behavior, and the second method involves adjusting cognitions to develop justifications for the addictive behavior. The second method is evidently preferred. Therefore, this model may provide theoretical support for prevention and remediation plans for IA among Chinese students at the very beginning of their college years.

## Limitations and Future Directions

Several limitations of this study are worth noting. First, although we proposed a theoretical model regarding the generation and development of IA, this model was not fully validated in the current study, and we focused on only the reciprocal relationship between IA and NMC. As such, further empirical research should be conducted to verify this model. Moreover, this theoretical model might have failed to address the influence of several other factors such as emotion and external environment. Therefore, more sophisticated theoretical models should be developed to address this issue. Second, to explore the reciprocal relationship between IA and NMC, this study conducted three surveys from September 2015 to January 2016. However, the time span of the three surveys may have been too short to detect a stable change in IA over time. The development trend may possibly be distinct at subsequent periods during the students’ college lives. Hence, further exploration of this issue in the remaining years of their time at college is necessary. Finally, the use of a convenient sample of college freshmen in this study was necessary because of constraints involving finances and human resources. This sample involved only 213 participants, all of whom came from university in Shandong province, China. Economic and cultural differences between provinces may affect the relationships between the two key variables involving college freshmen. Therefore, the study should be replicated with a larger sample involving different regions of China.

## Author Contributions

PH contributed to the initial idea conception, and the writing of manuscript. PW and FG contributed to the critical revisions. QL and YT helped to complete the data collection and analysis. All authors approved the final version of the manuscript for publication.

## Conflict of Interest Statement

The authors declare that the research was conducted in the absence of any commercial or financial relationships that could be construed as a potential conflict of interest.

## References

[B1] Ahmadi K. Saghafi A. (2013). Psychosocial profile of Iranian adolescents’ internet addiction. *Cyberpsychol. Behav. Soc. Network.* 16 543–548. 10.1089/cyber.2012.0237 23614793

[B2] Ayas T. Horzum M. B. (2013). Relation between depression, loneliness, self-esteem and internet addiction. *Education* 133 183–190.

[B3] Bai Y. Fan F. M. (2005). A study on the internet dependence of college students: the revising and applying of a measurement. *Psychol. Dev. Educ.* 4 99–104. 10.3969/j.issn.1001-4918.2005.04.019

[B4] Bessière K. Kiesler S. Kraut R. Boneva B. S. (2008). Effect of internet use and social resources on changes in depression. *Inform. Commun. Soc.* 11 47–70. 10.1080/13691180701858851

[B5] Caplan S. (2010). Theory and measurement of generalized problematic internet use: a two-step approach. *Comput. Hum. Behav.* 26 1089–1097. 10.1016/j.chd.2010.03.012

[B6] Caplan S. E. Turner J. S. (2007). Bringing theory to research on computer mediated comforting communication. *Comput. Hum. Behav.* 23 985–998. 10.1016/j.chb.2005.08.003

[B7] Chen S. K. (2012). Internet use and psychological well-being among college students: a latent profile approach. *Comput. Hum. Behav.* 28 2219–2226. 10.1016/j.chb.2012.06.029

[B8] Chiou W. B. Wan C. S. (2007). Using cognitive dissonance to induce adolescents’ escaping from the claw of online gaming: the roles of personal responsibility and justification of cost. *CyberPsychol. Behav.* 10 663–670. 10.1089/cpb.2007.9972 17927534

[B9] Chuang C. (2006). Massively multiplayer online role-playing game-induced seizures: a neglected health problem in internet addiction. *Cyberpsychol. Behav.* 9 451–456. 10.1089/cpb.2006.9.451 16901249

[B10] Cooper J. (2007). *Cognitive Dissonance: 50 Years of a Classic Theory*. London: Sage.

[B11] Cotten S. R. (2008). Students’ technology use and the impacts on well-being. *New Dir. Stud. Serv.* 124 55–70. 10.1002/ss.295

[B12] Daniel L. K. Paul H. D. Mark D. G. Michael G. (2012). Cognitive-behavioral approaches to out patient treatment of internet addiction in children and adolescents. *J. Clin. Psychol.* 68 1185–1195. 10.1002/jclp.21918 22976240

[B13] Davis R. A. (2001). A cognitive behavioural model of pathological internet use. *Comput. Hum. Behav.* 17 187–195. 10.1016/S0747-5632(00)00041-8

[B14] de Vries J. Timmins F. (2016). Care erosion in hospitals: problems in reflective nursing practice and the role of cognitive dissonance. *Nurse Educ. Today* 38 5–8. 10.1016/j.nedt.2015.12.007 26733428

[B15] Festinger L. (1957). *A theory of cognitive dissonance.* Evanston, IL: Row, Peterson.

[B16] Forrest C. J. King D. L. Delfabbro P. H. (2016). The measurement of maladaptive cognitions underlying problematic video-game playing among adults. *Comput. Hum. Behav.* 55 399–405. 10.1016/j.chb.2015.09.017

[B17] Forrest C. J. King D. L. Delfabbro P. H. (2017). Maladaptive cognitions predict changes in problematic gaming in highly-engaged adults: a 12-month longitudinal study. *Addict. Behav.* 65 125–130. 10.1016/j.addbeh.2016.10.013 27816037

[B18] Fu K. W. Chan W. S. Wong P. W. Yip P. S. (2010). Internet addiction: prevalence, discriminant validity and correlates among adolescents in Hong Kong. *Br. J. Psychiatry* 196 486–492. 10.1192/bjp.bp.109.075002 20513862

[B19] Georgios F. Konstantinos S. Ariadni S. Loannis G. Georgios G. (2014). The relationship between personality, defense styles, internet addiction disorder, and psychopathology in college students. *Cyberpsychol. Behav. Soc. Netw.* 17 6722–6676. 10.1089/cyber.2014.0182 25225916

[B20] Holbert R. L. Stephenson M. T. (2002). Structural equation modeling in the communication sciences, 1995-2000. *Hum. Commun. Res.* 28 531–551. 10.1111/j.1468-2958.2002.tb00822.x

[B21] Joseph C. P. Philip P. Baljinder S. Sarah M. Chris J. Andrew T. G. (2016). The development of compulsive internet use and mental health: a four-year study of adolescence. *Dev. Psychol.* 52 272–283. 10.1037/dev0000070 26595355

[B22] Kalkan M. (2012). Productiveness of interpersonal cognitive distortions on university students’ problematic internet use. *Child. Youth Serv. Rev.* 34 1305–1308. 10.1016/j.childyouth.2012.03.003

[B23] Kim E. J. Namkoong K. Ku T. Kim S. J. (2008). The relationship between online game addiction and aggression, self-control and narcissistic personality traits. *Eur. Psychiatry* 23 212–218. 10.1016/j.eurpsy.2007.10.010 18166402

[B24] King D. L. Delfabbro P. H. (2014). The cognitive psychology of Internet gaming disorder. *Clin. Psychol. Rev.* 34 298–308. 10.1016/j.cpr.2014.03.006 24786896

[B25] King D. L. Delfabbro P. H. (2016). The cognitive psychopathology of internet gaming disorder in adolescence. *J. Abnormal Child Psychol.* 44 1635–1645. 10.1007/s10802-016-0135-y 26875565

[B26] Kraut R. Kiesler S. Boneva B. Cummings J. Helgeson V. Crawford A. (2002). Internet paradox revisited. *J. Soc. Issues* 58 49–74. 10.1111/1540-4560.00248

[B27] Li D. L. Zhang W. Wang Y. H. Li D. P. (2013). Maternal psychological control and adolescents’ problematic internet use: the mediating role of maladaptive cognition. *Psychol. Sci.* 36 411–416.

[B28] Li H. Wang S. (2013). The role of cognitive distortion in online game addiction among Chinese adolescents. *Child. Youth Serv. Rev.* 35 1468–1475. 10.1016/j.childyouth.2013.05.021

[B29] Li N. Liang N. J. (2007). A study on the cognitive foundation of undergraduates’ internet addiction disorder. *Psychol. Sci.* 30 65–68. 10.3969/j.issn.1671-6981.2007.01.015

[B30] Liu G. C. Jen J. Y. Chen C. Y. Yen C. F. Chen C. S. Lin W. C. (2014). Brain activation for response inhibition under gaming cue distraction in internet gaming disorder. *Kaohsiung J. Med. Sci.* 30 43–51. 10.1016/j.kjms.2013.08.005 24388058 PMC11916293

[B31] Liu Q. X. Fang X. Y. Deng L. Y. Zhang J. T. (2012). Parenteadolescent communication, parental internet use and internet-specific norms and pathological internet use among Chinese adolescents. *Comput. Hum. Behav.* 28 1269–1275. 10.1016/j.chb.2012.02.010

[B32] Lu X. Yeo K. J. (2015). Pathological internet use among Malaysia university students: risk factors and the role of cognitive distortion. *Comput. Hum. Behav.* 45 235–242. 10.1016/j.chb.2014.12.021

[B33] Mai Y. Hu J. Yan Z. Zhen S. Wang S. Zhang W. (2012). Structure and function of maladaptive cognitions in pathological internet use among Chinese adolescents. *Comput. Hum. Behav.* 28 2376–2386. 10.1016/j.chb.2012.07.009

[B34] Mike Z. Y. He J. Deborah M. K. Pang K. C. (2014). The influence of personality behaviors, and self-esteem on internet addiction: a study of chinese college students. *Cyberpsychol. Behav. Soc. Network.* 17 104–110. 10.1089/cyber.2012.0710 PMC3924803 24003966

[B35] Mike Z. Y. Zhong Z. J. (2014). Loneliness, social contacts and internet addiction: a cross-lagged panel study. *Comput. Hum. Behav.* 30 164–170. 10.1016/j.chb.2013.08.007

[B36] Müller K. W. Glaesmer H. Brähler E. Woelfling K. Beutel M. E. (2014). Prevalence of internet addiction in the general population: results from a German population-based survey. *Behav. Inform. Technol.* 33 757–766. 10.1080/0144929X.2013.810778

[B37] Ni X. L. Yan H. Chen S. L. Liu Z. G. (2009). Factors influencing internet addiction in a sample of freshmen university students in China. *Rapid Commun.* 12 327–330. 10.1089/cpb.2008.0321 19445631

[B38] Peng W. Liu M. (2010). Online gaming dependency: a preliminary study in China. *Cyberpsychol. Behav. Soc. Network.* 13 329–333. 10.1089/cyber.2009.0082 20557254

[B39] San C. (2015). The CNNIC issued the thirty-fifth china internet development statistics report. *Dis. Educ. China* 4 99–104. 10.13541/j.cnki.chinade.2015.02.006

[B40] Shaw M. Black D. W. (2008). Internet addiction: definition, assessment, epidemiology and clinical management. *CNS Drugs* 22 353–365. 10.2165/00023210-200822050-00001 18399706

[B41] Shek D. T. L. Sun R. C. F. Yu L. (2013). “Internet addiction,” in *Neuroscience in the 21st Century: From Basic to Clinical*, ed. Pfaff D. W. (New York, NY: Springer), 2775–2811. 10.1007/978-1-4614-1997-6_108

[B42] Tian Y. Bian Y. L. Han P. G. Gao F. Q. Wang P. (2017). Associations between psychosocial factors and generalized pathological internet use in Chinese University students: a longitudinal cross-lagged analysis. *Comput. Hum. Behav.* 72 178–188. 10.1016/j.chb.2017.02.048 30376647

[B43] Tian Y. Bian Y. L. Han P. G. Wang P. Gao F. Q. (2015). The effect of shyness on internet addiction: the mediating effects of immersion tendency and network-related maladaptive cognition. *Chin. J. Spec. Educ.* 12 83–89. 10.3969/j.issn.1007-3728.2015.12.014

[B44] Tokunaga R. S. Rains S. A. (2010). An evaluation of two characterizations of the relationships between problematic internet use, time spent using the internet, and psychosocial problems. *Hum. Commun. Res.* 36 512–545. 10.1111/J.1468-2958.2010.01386.X

[B45] Tsai H. F. Cheng S. H. Yeh T. L. Shih C. C. Chen K. C. Yang Y. C. (2009). The risk factor of internet addiction-a survey of university freshmen. *Psychiatry Res.* 167 294–299. 10.1016/j.psychres.2008.01.015 19395052

[B46] Van den Eijnden R. J. J. M. Spijkerman R. Vermulst A. A. Van Rooij T. J. Engles R. C. M. E. (2010). Compulsive internet use among adolescents: bidirectional parent-child relationships. *J. Abnorm. Child Psychol.* 38 77–89. 10.1007/s10802-009-9347-8 19728076 PMC2809946

[B47] Van Lier P. A. Vitaro F. Barker E. D. Brendgen M. Tremblay R. E. Boivin M. (2012). Peer victimization, poor academic achievement, and the link between childhood externalizing and internalizing problems. *Child Dev.* 83 1775–1788. 10.1111/j.1467-8624.2012.01802.x 22716904

[B48] Wang T. Wei H. Zhou Z. K. Xiong J. Li X. Yang X. (2015). Relationships of peer player proportion, maladaptive cognition, and online game addiction. *Chin. J. Clin. Psychol.* 23 487–493. 10.16128/j.cnki.1005-3611.2015.03.023

[B49] Wen Z. L. Chang L. Hou J. T. (2006). Mediated moderator and moderated mediator. *Acta Psychol. Sin.* 38 448–452.

[B50] Woodhouse S. S. Dykas M. J. Jude C. (2012). Loneliness and peer relations in adolescence. *Rev. Soc. Dev.* 21 273–293. 10.1111/j.1467-9507.2011.00611.x

[B51] Yang L. S. Sun L. Zhang Z. H. Sun Y. H. Wu H. Y. Ye D. Q. (2014). Internet addiction, adolescent depression, and the mediating role of life events: Finding from a sample of Chinese adolescents. *Int. J. Psychol.* 49 342–347. 10.1002/ijop.12063 25178955

[B52] Young K. S. de Abreu C. N. (2011). *Internet Addiction: A Handbook and Guide to Evaluation and Treatment.* Hoboken, NJ: Wiley.

[B53] Young K. S. Pistner M. O’Mara J. Buchanan J. (1999). Cyber-disorders: the mental health concern for the new millennium. *Cyberpsychol. Behav.* 2 475–479. 10.1089/cpb.1999.2.475 19178220

[B54] Yuan Y. C. Shao A. H. Liang L. C. Bian Y. F. (2014). A cross-lagged analysis of the relation between unsociability, peer rejection and peer victimization. *Psychol. Dev. Educ.* 30 16–23.

[B55] Zhang H. Y. Li D. P. Li X. (2015). Temperament and problematic internet use in adolescents: a moderated mediation model of maladaptive cognition and parenting styles. *J. Child Family Stud.* 24 1886–1897. 10.1007/s10826-014-9990-8

[B56] Zhang J. T. Chen C. Wang L. J. Liu L. Liu F. E. Zhao H. C. (2014). The relationship between time spent online and internet addiction among chinese college freshmen: a mediated moderation model. *Acta Psychol. Sin.* 30 65–68. 10.3969/j.issn.1671-6981.2007.01.015

[B57] Zhou Z. Yuan G. Yao J. (2012). Cognitive biases toward Internet game-related pictures and executive deficits in individuals with an Internet game addiction. *PLoS ONE* 7:e48961 10.1371/journal.pone.0048961 PMC3498351 23155434

